# The effects of age on clinical characteristics, hospitalization and mortality of patients with influenza‐related illness at a tertiary care centre in Malaysia

**DOI:** 10.1111/irv.12691

**Published:** 2020-02-05

**Authors:** Pui Li Wong, Hoe Leong Sii, Chun Keat P'ng, Soon Sean Ee, Xiang Yong Oong, Kim Tien Ng, Nik Sherina Hanafi, Kok Keng Tee, Maw Pin Tan

**Affiliations:** ^1^ Department of Medicine Faculty of Medicine University of Malaya Kuala Lumpur Malaysia; ^2^ Department of Primary Care Medicine Faculty of Medicine University of Malaya Kuala Lumpur Malaysia; ^3^ Department of Medical Microbiology Faculty of Medicine University of Malaya Kuala Lumpur Malaysia; ^4^ Department of Medical Sciences School of Healthcare and Medical Sciences Sunway University Petaling Jaya Malaysia

**Keywords:** age differences, clinical characteristics, elderly, influenza, mortality, South‐East Asia

## Abstract

**Background:**

Age is an established risk factor for poor outcomes in individuals with influenza‐related illness, and data on its influence on clinical presentations and outcomes in the South‐East Asian settings are scarce. The aim of this study was to determine the above among adults with influenza‐related upper respiratory tract infection at a teaching hospital in Malaysia.

**Methods:**

A retrospective case‐note analysis was conducted on a cohort of 3935 patients attending primary care at the University Malaya Medical Centre, Malaysia from February 2012 till May 2014 with URTI symptoms. Demographics, clinical characteristics, medical and vaccination history were obtained from electronic medical records, and mortality data from the National Registration Department. Comparisons were made between those aged <25, ≥25 to <65 and ≥65 years.

**Results:**

470 (11.9%) had PCR‐confirmed influenza virus infection. Six (1.3%) received prior influenza vaccination. Those aged ≥65 years were more likely to have ≥2 comorbidities (*P* < .001) and were less likely to present with fever (*P* = .004). One‐third of those aged ≥65 years experienced hospitalization, intensive care admission or death within a year compared to 10% in the ≥25 to <65 years. Age ≥65 years was an independent predictor of hospitalization and death (OR = 9.97; 95% CI = 3.11‐31.93) compared to those aged <25 years.

**Conclusion:**

Older patients in our cohort were more likely to have comorbidities and present with atypical features, with older age being an independent predictor of poor health outcomes. Our findings will now inform future health policies on older persons and economic modelling of adult vaccination programmes.

## 1 INTRODUCTION

Influenza‐associated respiratory infection is recognized as a major public health concern globally.^1^ There are an estimated 300 000‐650 000 deaths annually due to seasonal influenza‐associated respiratory infections worldwide, with South‐East Asia having the second highest estimated mortality rates (EMR) of 3.5‐9.2 per 100 000 individuals, second to sub‐Saharan Africa (2.8‐16.5 per 100 000).^2^ These rates increase significantly in the population aged more than 75 years with rates of 51.3‐99.4 per 100 000 individuals.^2^ The estimated global influenza death from respiratory infections published in 2018 appears to be higher than a previous estimate of 148 000‐249 000 in 2013.^3^

Furthermore, the influenza virus is also implicated in a large number of outpatient visits and subsequent hospitalization, accounting for 10%‐26% of outpatient visits with influenza‐like illness (ILI) and 6%‐14% pneumonia hospitalization in East and South‐East Asia.^4^, ^5^, ^6^, ^7^ Children, older persons, individuals with chronic respiratory diseases and the immunocompromised are considered high‐risk groups for complications from influenza viral infections.^5^ Clinical manifestations have also been known to differ with age, as older adults are less likely to report classical ILI symptoms.^1^, ^8^ Nevertheless, fever, cough and acute illness onset remain predictive of influenza among older adults.^8^, ^9^

Malaysia is a middle‐income country in South‐East Asia with a population of 31 million and a gross national income (GNI) per capita of USD 9800 as of 2017.^10^ Influenza viral infections are usually seen year round in this country and typically peaks in the dry months of April to June, and wet months of October to January^11^ with surveillance data from 2007 to 2013 showing influenza A virus as the predominant type, and influenza B virus accounting for 28.9% of cases.^12^ Due to this pattern, influenza vaccinations are given all year round with Northern formulations in October and Southern formulations in April of each year.

Disability rates in older Malaysians are significantly higher at 16% compared to developed countries such as Canada which only reported a 6% disability rate when the same assessment tool for activities of daily living was employed.^13^ To our knowledge, there is no published report of vaccine uptake among older adults in Malaysia, but vaccine sales figures from the H1N1 pandemic reported sales of 1408 per 100 000.^14^ Published data on clinical features and health outcomes associated with influenza infection in Malaysia remains scarce and even more so in older Malaysians with differing disability and vaccination rates.^15^ Therefore, we aimed to examine differences in clinical characteristics, clinic visits, hospitalization and death in younger, middle‐aged and older Malaysians with laboratory‐confirmed influenza diagnosed during a primary care visit with upper respiratory tract symptoms.

## 2 METHODOLOGY

### 2.1 Study setting and population

This was a retrospective cohort study based on a laboratory culture data set collected from February 2012 to May 2014 in University of Malaya Medical Centre (UMMC), a tertiary teaching hospital in Kuala Lumpur, Malaysia. The study had received ethical approval from the local institutional review board (MREC ID: 2016714‐4027).

A total of 3935 patients who presented to the primary care clinic during the period of the study with symptoms associated with URTI (sneezing, nasal discharge, nasal obstruction, headache, sore throat, hoarseness of voice, myalgia and cough) were recruited. Nasopharyngeal swab samples were then obtained and transported in universal transport medium to the laboratory for reverse transcription polymerase chain reaction (RT‐PCR) for detection of respiratory viruses. The study methods are described in an earlier article published elsewhere.^16^ Individuals who tested positive for influenza viruses were included in this study.

### 2.2 Clinical data collection

Sociodemographic and clinical data including age, gender, ethnicity, duration of illness at presentation, clinical features, comorbidities, smoking history, vaccination history, subsequent outpatient visits and hospitalization were extracted from the hospital Electronic Medical Record system, using a standardized data collection document (Table S1). Within the primary care department, all patients will have blood pressure measured routinely using automated oscillometric blood pressure monitors on arrival by the clinic nurse prior to their consultation. The blood pressure measurements were then recorded to the nearest mm Hg using an Electronic Medical Record template. Vital status and dates of death were obtained from the National Registration Department.

### 2.3 Study instruments

Total nucleic acids (NA) from nasopharyngeal swab samples were extracted using the NucliSENS easyMAG automated nucleic acid extraction system (BioMérieux).^17^ The xTAG Respiratory Virus Panel (RVP) FAST multiplex RT‐PCR assay (Luminex Molecular Diagnostics, USA) was then used to detect the influenza viruses (A and B) and other respiratory viruses.^18^ The procedures above were performed according to the manufacturer's protocol.

### 2.4 Data analysis

The sample size of the study was based on all consecutive patients presenting with ILI to the primary care department of the teaching hospital within the two year period, the minimal period required to study seasonal changes. A retrospective calculation was conducted to determine the power of this study. Assuming that 10% of individuals <65 years of age were either hospitalized or had died within 1 year of presentation, a total sample size of 238 patients will provide 80% power to detect a trebling in relative risk.

The sample population was divided into three age groups: Group 1 (<25 years), Group 2 (25 years‐64 years) and Group 3 (65 years and over), to allow for direct comparisons with global influenza surveillance reports.^19^ The age group of 0‐14 was excluded as this study only involved adults. Data were presented as frequencies with percentages in parentheses, mean with standard deviation in parentheses or median with interquartile ranges where appropriate. The independent Student's *t* test, Mann‐Whitney *U*, chi‐squared and Fisher's exact tests were used where appropriate for group comparisons in sociodemographic, number of comorbidities, clinical characteristics and outcomes of influenza‐associated infection across different age groups. Logistic regression analyses were then conducted to determine the influence of age on clinical outcomes of influenza‐associated infection using a composite variable of hospital admission, with or without intensive care unit (ICU) admission or deaths (underlying influenza, pneumonia, respiratory or circulatory deaths), after separate adjustments for potential confounders, such as number of comorbidities and presence of chronic conditions, and the presence of fever. In this analysis, Group 1 was considered the reference category, and comparisons were made between Group 2 and Group 1, and Group 3 and Group 1.

## 3 RESULTS

### 3.1 Sociodemographics

Of the 3935 samples collected during the study period, samples from 470 (11.9%) patients with ILI were found to be RT‐PCR positive for influenza infection. Only data from 450 individuals were included in the final analysis due to incomplete data. 277 (61.6%) samples tested positive for the influenza A virus and 173 (38.4%) for the influenza B virus. Of the 450 patients, 245 (52.1%) were female. One hundred and five (23.3%) were aged <25 years, 292 (64.9%) were aged 25‐64 years, and 53 (11.8%) were aged 65 years or greater. There were differences in ethnicity, number of comorbidities, diagnoses of diabetes, hypertension, viral hepatitis, chronic kidney disease, cardiovascular disease and malignancy between groups (Table 1).

**Table 1:** Baseline sociodemographics and clinical characteristics

Variable	All	Age (y)			*P* value
		<25	≥25 to <65	≥65	
Number of patients, (percentage)	450	105 (23.3)	292 (64.9)	53 (11.8)	–
Number of influenza vaccination	6 (1.3)	1 (1.0)	5 (1.7)	–	–
Gender					.623
Male	205 (45.6)	50 (47.6)	134 (45.9)	21 (39.6)	
Female	245 (54.4)	55 (52.4)	158 (54.1)	32 (60.4)	
Ethnicity					**<.001**
Malays	194 (43.1)	58 (55.2)	118 (40.4)	18 (34.0)	
Chinese	94 (20.9)	16 (15.2)	55 (18.8)	23 (43.4)	
Indians	143 (31.8)	28 (26.7)	105 (36.0)	10 (18.9)	
Others^a^	19 (4.2)	3 (2.9)	14 (4.8)	2 (3.8)	
Number of comorbidities					**<.001**
None	245 (54.4)	87 (82.9)	149 (51.0)	9 (17.0)	
1	73 (16.2)	5 (4.8)	54 (18.5)	14 (26.4)	
≥2	132 (29.3)	13 (12.4)	89 (30.5)	30 (56.6)	
Chronic conditions					
Diabetes mellitus	90 (20.0)	3 (2.9)	62 (21.2)	25 (47.2)	**<.001**
Hypertension	117 (26.0)	–	80 (27.4)	37 (69.8)	**<.001**
Asthma/reactive airway disease	45 (10.0)	8 (7.6)	34 (11.6)	3 (5.7)	.266
Autoimmune disease	12 (2.7)	1 (1.0)	10 (3.4)	1 (1.9)	.375
Haematological disorders	–	–	–	–	–
Chronic obstructive airway disease	–	–	–	–	–
Chronic liver disease	–	–	–	–	–
Viral hepatitis	8 (1.8)	–	5 (1.7)	5 (1.7)	**.039**
Chronic kidney disease	8 (1.8)	–	5 (1.7)	3 (5.7)	**.039**
Cardiovascular disease	41 (9.1)	1 (1.0)	25 (8.6)	15 (28.3)	**<.001**
Immunosuppression	2 (0.4)	–	2 (0.7)	–	.581
HIV	–	–	–	–	–
Malignancy	20 (4.4)	1 (1.0)	12 (4.1)	7 (13.2)	**.002**
Obesity	16 (3.6)	3 (2.9)	12 (4.1)	1 (1.9)	.657
Obstructive sleep apnoea	4 (0.9)	1 (1.0)	3 (1.0)	–	.762
Steroid usage (in asthma)	38 (8.4)	6 (5.7)	29 (9.9)	3 (5.7)	.579
Smoking					.418
Yes	25 (5.6)	5 (4.8)	15 (5.1)	5 (9.4)	
No	425 (90.4)	100 (95.2)	277 (94.9)	48 (90.6)	
Any medication prescription					.143
Yes	383 (84.9)	84 (80.0)	255 (87.3)	43 (81.1)	
No	68 (15.1)	21 (20.0)	37 (12.7)	10 (18.9)	

a Indigenous people of East Malaysia, Sikhs, Bangladeshi, Indonesians, Filipinos, Sudanese and Pakistanis

Smoking was only reported in 5.6% of our sample. Only six (1.3%) patients had a history of influenza vaccination, with only one of them being in the at‐risk group. No individual over the age of 65 years had previously received any influenza vaccination.

### 3.2 Clinical characteristics and age

The clinical symptoms and blood pressure of all included cases, according to the three age categories are summarized in Table 2. Four hundred and twenty‐four (94.2%) of all cases presented within 7 days of symptom onset. There was no difference in duration of illness with age categories. Cough (84.7%), 63.6% of which was productive, and fever (81.3%) were the most commonly reported clinical symptoms. Nasal discharge and sore throat were reported in 59.6% and 45%, respectively.

**Table 2:** Clinical features by age groups

Variable	All	Age (y)			*P* value
		<25	≥25 to <65	≥65	
Duration of illness (d)					.260
<7	424 (94.2)	100 (95.2)	277 (94.9)	47 (88.7)	
7‐14	23 (5.1)	4 (3.8)	13 (4.5)	6 (11.3)	
>14	3 (0.7)	1 (1.0)	2 (0.7)	–	
Clinical symptoms					
Fever	366 (81.3)	96 (91.4)	232 (79.5)	38 (71.1)	.004
Chill or rigor	36 (8.0)	10 (9.5)	24 (8.2)	2 (3.8)	.441
Cough	381 (84.7)	84 (80.0)	248 (84.9)	49 (92.5)	.119
Productive cough	286 (63.6)	64 (61.0)	186 (63.7)	36 (67.9)	.688
Sore throat	197 (43.8)	49 (46.7)	129 (44.2)	19 (35.8)	.421
Retroorbital pain	4 (0.9)	4 (3.8)	–	–	.001
Headache	48 (10.7)	17 (16.2)	30 (10.3)	1 (1.9)	.021
Myalgia	89 (19.8)	22 (21.0)	60 (20.5)	7 (13.2)	.440
Joint pain	24 (5.3)	8 (7.6)	15 (5.1)	1 (1.9)	.308
Nasal discharge	268 (59.6)	69 (65.7)	169 (57.9)	30 (56.6)	.335
Nasal congestion	179 (39.8)	49 (46.7)	111 (38.0)	19 (35.8)	.246
Sneezing	68 (15.1)	19 (18.1)	46 (15.8)	3 (5.7)	.105
Conjunctivitis	5 (1.1)	3 (2.9)	–	2 (3.8)	.008
Lymphadenopathy	5 (1.1)	3 (2.9)	2 (0.7)	–	.136
Lethargy	15 (3.3)	3 (2.9)	11 (3.8)	1 (1.9)	.745
Rash	5 (1.1)	–	5 (1.7)	–	.255
Reduced oral intake	21 (4.7)	8 (7.6)	8 (2.7)	5 (9.4)	.027
Reduced appetite	32 (7.1)	9 (8.6)	19 (6.5)	4 (7.5)	.773
Hoarseness of voice	18 (4.0)	5 (4.8)	13 (4.5)	–	.283
Shortness of breath	12 (2.7)	1 (1.0)	8 (2.7)	3 (5.7)	.220
Shortness of breath (on exertion)	12 (2.7)	1 (1.0)	7 (2.4)	4 (7.5)	.047
Systolic blood pressure, mm Hg					<.001
<100	4 (0.9)		1 (0.3)	–	
100‐119	106 (23.6)	3 (2.9)	61 (20.9)	7 (13.2)	
120‐140	195 (43.3)	38 (36.2)	145 (49.7)	24 (45.3)	
>140	26 (5.8)	26 (24.8)	15 (5.1)	11 (20.8)	
Diastolic blood pressure, mm Hg					.049
<80	176 (39.3)	44 (41.9)	115 (39.5)	17 (32.7)	
80‐89	122 (27.2)	20 (19.0)	85 (29.2)	17 (32.7)	
90‐100	23 (5.1)	3 (2.9)	14 (4.8)	6 (11.5)	
>100	5 (1.1)	–	4 (1.4)	1 (1.9)	

Differences in the proportion of cases with fever, headache, reduced oral intake, systolic and diastolic blood pressure exist between groups. Ninety‐six of the 105 (91.4%) individuals aged <25 years had symptoms of fever, compared to 232/292 (79.5%) in those aged 25‐64 years, and 38/53 (71.1%) of those aged 65 years and over (*P* = .004). Headache was reported by 17 (16.2%) of under 25 seconds, 30 (10.3%) of those aged 25‐64 years and one (1.9%) of those aged 65 years and over (*P* = .021). Reduced oral intake was present in eight (7.6%) of under 25 seconds, eight (2.7%) of those aged 25‐64 years and five (9.4%) of those aged 65 years and over (*P* = .027). Forty‐one (39.1%) individuals aged below 25 years had a systolic blood pressure of 119 mm Hg and below, compared to only seven (13.2%) of those aged 65 years and over. Forty‐four (41.9%) individuals aged below 25 had a diastolic blood pressure below 80 mm Hg compared to 17 (32.7%) of individuals aged 65 years and over. Those aged ≥65 years were more likely to have ≥2 comorbidities (*P* < .001).

### 3.3 Clinical outcomes

Table 3 summarizes the number of subsequent primary care visits with similar URTI and hospital admission or death within 1 year after the episode of PCR‐diagnosed influenza infection. Ninety‐seven of the 450 (21.6%) cases with influenza presented to the outpatient clinic with a second URTI episode within a year after initial presentation. There was no difference in subsequent visits with URTI with age categories. Five deaths (1.1%) occurred in the overall sample of 450 confirmed influenza cases within a year.

**Table 3:** Subsequent primary care visit and hospital admissions by age groups

Variable	All	Age (years)			*P*‐value
		<25	≥25 to <65	≥65	
Total number of subsequent visit to primary care with similar URTI within 1 y					.174
1 visit	97 (21.6)	24 (23.1)	54 (18.5)	19 (35.8)	
2 visits	16 (3.6)	4 (3.8)	11 (3.8)	1 (1.9)	
3 visits	5 (1.1)	–	5 (1.7)	–	
4 visits	4 (0.8)	–	4 (1.4)	–	
None	325 (72.2)	74 (71.2)	218 (74.7)	33 (62.3)	
Hospital admission (with or without ICU admission) or death within one year^a^	47 (10.4)	4 (3.8)	28 (9.6)	15 (28.3)	<.001

Abbreviations: ICU, intensive care unit; URTI, upper respiratory tract infection.

a Death is defined as underlying influenza, pneumonia, respiratory or circulatory deaths.

Hospital admission (with or without ICU admission) or death was reported in 47 (10.4%) of the overall sample. Fifteen of the 53 (28.3%) individuals in the aged 65 years and over age group were admitted to hospital or died within 1 year compared to four individuals in the <25 years age group and 28 (9.6%) of those aged 25‐64 years (*P* < .001). All five deaths occurred in those aged 65 years and over.

### 3.4 Logistic Regression

The odds ratios (OR) and 95% confidence intervals (CI) examining the association between the composite outcome of hospitalization and death are presented in Table 4. As mentioned earlier, comparisons between groups were made using dummy variables with the under 25 age group as the reference category. First, the unadjusted models followed by models adjusted for number of comorbidities, diabetes, hypertension and cardiovascular disease (Model 1) and number of comorbidities, diabetes, hypertension, cardiovascular disease and fever (Model 2) were presented. The risk of hospitalization or death in individuals in the ≥65 years age group was significantly greater compared to individuals aged <25 years (OR = 9.96; 95% CI = 3.11‐31.93). This remained significant following adjustments for number of comorbidities, diabetes, hypertension and cardiovascular disease (OR = 4.46; 95%CI = 1.21‐16.47) and additional adjustments for the presence of fever (OR = 4.24; 95%CI = 1.15‐15.67).

**Table 4:** Logistic regression models for hospitalization and death according to age groups

Age group (years)	Unadjusted		Model 1^a^		Model 2^b^	
	OR (95% CI)	*P* value	OR (95% CI)	*P* value	OR (95% CI)	*P* value
<25	1		1		1	
≥25 to <65	2.68 (0.92, 7.83)	.072	1.81 (0.59, 5.54)	.301	1.73 (0.56, 5.34)	.339
≥65	9.97 (3.11, 31.93)	<.001	4.46 (1.21, 16.47)	.025	4.24 (1.15, 15.67)	.031

Abbreviations: CI, confidence interval; OR, odds ratio.

a Model 1: Adjusted for potential confounders: number of comorbidities, chronic conditions (diabetes mellitus, hypertension, cardiovascular disease).

b Model 2: Adjusted for all factors in Model 1 and presence of fever.

## 4 DISCUSSION

Influenza is a major infectious disease that causes substantial morbidity and mortality worldwide every year. Better understanding of the disease burden among clinicians could lead to increased uptake of appropriate vaccinations in high‐risk groups and reduce the cases of influenza infection, particularly in South‐East Asian countries such as Malaysia, where awareness towards influenza vaccination remains low.^14^ In this study, we evaluated the influence of age on clinical features and outcomes of influenza infection particularly among a mostly unvaccinated group with a 1% influenza vaccine uptake.

Our findings overall showed that cough is the most common clinical finding, accounting for approximately 85% of cases whereas fever and nasal congestion were present in >80% and >50% of cases, respectively. These features are consistent with the World Health Organization's (WHO) definition of ILI and previous observations.^20^, ^21^ Symptoms of influenza typically subside within 5‐8 days^22^ with 95% of cases in this study reporting a duration of illness of less than a week.

Clinical manifestations of influenza have been known to vary with age. Our study found that fever occurred less commonly among cases aged 65 years and above compared to the younger and middle‐aged age groups. These findings are consistent with those of other studies looking at clinical characteristics of ILI in the older person. The study by Matsuno et al found that older patients aged 75 years and above have significantly lower body temperature than younger individuals with influenza‐associated pneumonia.^23^ Another study also found that lowering the triage temperature to 37.2°C increased the specificity of predicting influenza from 75% to 79%.^9^ The composite variable of self‐reported fever and a temperature of ≥37.2°C had the highest sensitivity of 76% with a negative predictive value of 95% and positive predictive value (PPV) of 20%.^9^ Fever, cough and acute onset of illness had a 30% PPV for older patients with influenza infection compared to a 70% PPV for younger patients.^24^ Absence of fever in older patients (which accounted for 30% of our older patients) may lead to misdiagnosis and delay in treatment which increases the risk of serious pulmonary complications.^25^ If the commonly used threshold of fever in ILI of 37.8°C were used for older persons, it would result in a sensitivity of only 57% and specificity of 71%.^26^

These findings may be due to the effect of immunosenescence in the older persons which affects both the innate and adaptive immune response,^27^ thus reducing the prevalence of fever in this age group. This should prompt us to consider revising the definition of ILI in older persons. It is important to note that although clinical features help in identifying patients with ILI, there is no single clinical finding that could confirm or exclude the diagnosis of influenza, and epidemiological data may be more useful in aiding management decisions.^28^

The composite outcome of hospitalization and all‐cause mortality was significantly higher in older patients. Nicholson et al also found significant morbidity in older patients with viral URTIs which led to increasing disease burden 29 and increased mortality.^30^ Individuals in the 65 years and older group in our study were significantly more likely to experience hospitalization or death in the subsequent year after adjustment for potential confounders. High hospitalization rates of 55.6 hospitalizations per 10 000 individuals have also been reported among unvaccinated older persons previously.^31^ A study by Ang et al^32^ on influenza‐associated hospitalizations showed that rates of hospitalization in those aged 75 years or older were 46 times higher than those aged 25‐44 years. A recent study estimated that the mean annual influenza‐associated respiratory EMR is higher at 2.9‐223.5 per 100 000 individuals for people aged above 65 years, in contrast to 0.1‐6.4 per 100 000 individuals for people younger than 65 years.^2^ Age‐specific estimates on influenza‐associated mortality extracted from the US national viral surveillance data indicated that 90% of influenza‐associated deaths occurred among persons aged 65 years or older with underlying respiratory and circulatory deaths.^30^ The latest report from the CDC from the 2017 to 2018 flu season shows that 70% of 959 000 hospitalized patients were aged 65 years or older, and they also accounted for 90% of 79 400 deaths compared to 10 300 among working age adults aged 18‐64 years.^33^ These staggering numbers support the call for vaccination and public health policies, and the development of better vaccines that offer enhanced protection for older persons.^34^

The likelihood of concurrent comorbidities and chronic conditions increases with age.^35^, ^36^, ^37^ A study by Mori et al^38^ suggested that there are potential confounders that may affect the results of studies looking at vaccine efficacy, and chronic illnesses are an example of one such factors. Groenwold et al^36^ also adjusted for confounders such as comorbidities in their study on influenza vaccination and mortality in older adults. Multivariate regression modelling is a common method used in observational studies on influenza to adjust for confounders. This provides alternative evidence on the burden of disease, as randomized controlled trials evidence on influenza which includes individuals aged 65 years and over are surprisingly lacking.^39^

The overall poor uptake of influenza vaccination by our study population and the older age group is apparent. Influenza vaccination has been shown to be beneficial to older community‐dwellers and nursing home residents.^40^, ^41^ It also reduces the rate of hospitalization for pneumonia and influenza by 48%‐57%, and for all respiratory conditions by 27%‐39%.^40^ While national guidelines for adult vaccinations do exist,^42^ government, insurer or employer funded adult vaccination programmes currently do not. Despite free health care for older persons in public health facilities,^43^ the annual influenza vaccination is funded out‐of‐pocket, with low awareness for the need to vaccinate, among healthcare workers and patients in high‐risk groups. This highlights the reactive nature of the current health service, with lack of emphasis on preventive measures.^44^ Therefore, the findings of this study will provide valuable information for economic modelling and to inform future policy makers on future decisions in preventive healthcare funding.

The findings of this study were limited by its retrospective design with data collection based on prior documentation. Hence, if symptoms were not documented, they were assumed absent, potentially underestimating symptom burden. In the analysis of our data, we have included all patients with influenza‐positive results, including those with co‐infections with other viruses. Where multiple viruses were detected, it would not be possible to discern if the virus was causal or non‐causal. Assumptions were also made that patients who did not have any documented influenza vaccinations in our electronic medical records, were unvaccinated. Lastly, all patients were recruited from a single site, and therefore, the findings of this study could not be generalized to the rest of population.

## 5 CONCLUSION

Older patients with influenza URTI in our cohort were less likely to present with fever, with an increased risk of the adverse outcomes of hospitalization or death. Uptake of influenza vaccination is 1% overall and 0% in the older age group. Future studies should evaluate this relationship prospectively in a representative sample and should also consider evaluating the benefits of vaccination in reducing healthcare burden. Our findings can be used for economic modelling as well as to highlight the major health burden associated with influenza infection within a developing nation with an equatorial climate.
