# Supplementary material for: The effects of age on clinical characteristics, hospitalization and mortality of patients with influenza‐related illness at a tertiary care centre in Malaysia
Source: Influenza Other Respir Viruses. 2020 Feb 5;14(3):286–93. doi: 10.1111/irv.12691 (PMC7182601; doi:10.1111/irv.12691)
Supplement: Supplementary file 1 [file IRV-14-286-s001.docx]

| Study Variables | Description and categories |
| --- | --- |
| Gender | Sex of the patient at birth (1= Male, 2= Female). |
| Ethnicity | Malaysia is a country made up of three main ethnicities and we have grouped them accordingly (1= Malay, 2= Chinese, 3= Indian, 4= Others).  Others include: Sikh, Bumiputera, Melanau, Bidayuh, French, Bangladeshi, Indonesian, Filipino, Sudanese, Pakistani. |
| Age group | Age is divided into three catergories, with patients aged (0=<25 years, 1= ≥25 to <65 years, and 2= ≥65 years). |
| Duration of illness | Number of days patients have been unwell at presentation. These have been divided into three groups (1= <7 days, 2= 7-14 days, 3=>14 days). |
| Number of comorbidities | Number of comorbidities refers to the number of chronic conditions present in patients and have been divided into three categories (1= None, 2= 1 comorbiditiy, 3= ≥2 comorbidities) |
| Chronic conditions | Comorbidities include common chronic conditions (1= diabetes mellitus, 2= hypertension, 3= asthma or reactive airway disease, 4= haematological disorders, 5= chronic obstructive airway disease, 6= chronic liver disease, 7= viral hepatitis, 8= chronic kidney disease, 9= cardiovascular disease, 10= immunosuppression, 11= Human Immunodeficiency Virus (HIV), 12= malignancy, 13= obesity, 14= obstructive sleep apnoea, 15= steroid usage in asthma). |
| Smoking | Number of patients who smoke (1= Yes, 2= No) |
| Any medication prescription | Medications prescribed include those for symptomatic relief and antibiotics (1= Yes, 2= No) |
| Clinical symptoms | Symptoms of upper respiratory tract illness was listed (0= Absent, 1= Present) |
| Systolic blood pressure | Systolic blood pressures were divided into three categories with (1= <100mmHg indicating low blood pressure, 2= 100-119mmgHg indicating a normal blood pressure, 3= 120-140mmgHg indicating a borderline high blood pressure, and 4= >140mmHg indicating a high blood pressure).  Unknowns were excluded. |
| Diastolic blood pressure | Diastolic blood pressures were divided into three categories with (1= <80mmHg indicating low blood pressure, 2= 80-89mmgHg indicating a normal blood pressure, 3= 90-100mmgHg indicating a borderline high blood pressure, and 4= >100mmHg indicating a high blood pressure).  Unknowns were excluded. |
| Outcome variables |  |
| Total number of subsequent visits to primary care with similar URTI within one year | Number of visits to primary care after the first visit with symptoms of upper respiratory tract infection in the one year following the index visit ( 0= None, 1= 1 visit, 2= 2 visits, 3= 3 visits, 4=4 visits). |
| Hospital admission (with or without ICU admission) or death within one year | Number of hospital admissions including admissions to the Intensive Care Unit or death within one year of index visit to primary care (1= Yes, 2=No) |
